# Antimicrobial efficacy of a citric acid/hydrochloric acid blend, peroxyacetic acid, and sulfuric acid against *Salmonella* and background microbiota on chicken hearts and livers

**DOI:** 10.1111/1750-3841.17037

**Published:** 2024-03-27

**Authors:** Emma Nakimera, Leslie Pearl M. Cancio, Gary A. Sullivan, Raziya Sadat, Byron D. Chaves

**Affiliations:** ^1^ Department of Food Science and Technology University of Nebraska‐Lincoln Lincoln Nebraska USA; ^2^ Department of Science and Technology (DOST) XI Technical Services Division Davao Philippines; ^3^ Department of Animal Science University of Nebraska‐Lincoln Lincoln Nebraska USA

**Keywords:** antimicrobials, chicken, offal, Salmonella, variety meats

## Abstract

This study aimed at evaluating the efficacy of a blend of citric acid and hydrochloric acid (CP), peroxyacetic acid (PAA), and sulfuric acid (SA) against *Salmonella* and mesophilic aerobic plate counts (APC) on chicken hearts and livers. Samples were inoculated with a five‐serovar cocktail of *Salmonella* at ca. 4.8 log CFU/g and treated by immersion with a water control (90 s), CP (5% v/v, 30 s), PAA (0.05% v/v or 500 ppm, 90 s), or SA (2% v/v, 30 s), all at 4°C and with mechanical agitation. Samples were vacuum packed and stored for up to 3 days at 4°C. Three independent replications were performed for each product, treatment, and time combination. The average *Salmonella* reductions in chicken hearts after 3 days were 1.33 ± 0.25, 1.40 ± 0.04, and 1.32 ± 0.12 log CFU/g for PAA, SA, and CP, respectively. For chicken livers, the values were 1.10 ± 0.12, 1.09 ± 0.19, and 0.96 ± 0.27 for PAA, SA, and CP, respectively. All antimicrobials reduced *Salmonella* counts in both chicken hearts and livers by more than one log, in contrast to the water control. All treatments effectively minimized the growth of APC for up to 3 days of refrigerated storage, and no differences in objective color values (L, a, or b) were observed. The poultry industry may use these antimicrobials as components of a multifaceted approach to mitigate *Salmonella* in nonconventional chicken parts.

## 1 INTRODUCTION

According to the Food and Agriculture Organization of the United Nations, poultry meat represents nearly 40% of the global meat production market, making it a significant commodity around the world (Food & Agricultural Organization of the United Nations, FAO, 2023). The United States of America is the world's largest producer of poultry meat, contributing 17% of the global output. China and Brazil follow as the second and third largest producers, respectively (FAO, 2023). Unfortunately, contaminated chicken products are one of the main vehicles for salmonellosis in humans (Centers for Disease Control & Prevention, CDC, 2023b). The CDC estimates that *Salmonella* is responsible for roughly 1.35 million infections, around 26,500 hospitalizations, and approximately 420 deaths annually in the U.S. alone (CDC, 2023a). Furthermore, outbreak data estimate that 17.3% of all foodborne salmonellosis cases in the U.S. are attributed to the consumption of contaminated chicken products (Interagency Food Safety Analytics Collaboration, 2022).

The large volume of broiler meat production in the U.S. implies that many nonconventional chicken products, such as hearts and livers, are inevitably produced. According to the Observatory of Economic Complexity (2022), the U.S. is the world's largest exporter of chicken gizzards and ranks third for chicken liver exports, after Brazil and Australia. These products fall under the “other poultry products” category established by the U.S. Department of Agriculture Food Safety and Inspection Service (USDA FSIS) to encompass items such as quarter‐carcasses, half‐carcasses, necks, hearts, gizzards, livers, and feet, and to differentiate them from more conventional products, in the U.S., such as chicken breasts, legs, thighs, and wings (USDA FSIS, 2020).

Contaminated chicken liver products have been linked to several outbreaks of *Salmonella* infections (Lanier et al., 2018). Jung et al. (2019) investigated the prevalence and concentration of *Salmonella* in retail chicken liver samples during a 9‐month period in the U.S. states of Delaware, New Jersey, and Pennsylvania. They found that 59.4% of chicken livers (148/249) were contaminated. The pathogen was also recovered significantly more frequently from livers packaged by retailers (66.9%, 81/121) than from livers packaged directly by processors (52.3%, 67/128), indicating a potential breach of sanitary practices at the point of sale (Jung et al., 2019). Studies outside of the U.S. have reported high prevalence rates for *Salmonella* in raw chicken livers. Procura et al. (2019) found that of 666 chicken liver samples collected from retail markets in Entre Ríos, Argentina, 32 (4.8%) were positive. In Iraq, the prevalence in imported frozen livers was 54.7% (52/95) (Al‐Rubaie et al., 2020). Additionally, the prevalence in chicken hearts has been reported as 35.7% (10/28) in imported frozen hearts in Iraq (Al‐Rubaie et al., 2020) and 48% (12/25) in Egypt (El‐Aziz, 2013).

Many U.S.‐approved antimicrobial agents can be employed in both initial and subsequent stages of chicken processing to minimize the presence of *Salmonella* and other pathogens on poultry carcasses and products (, USDA FSIS, 2022). Peroxyacetic acid (PAA) is a popular antimicrobial used by most harvest and processing establishments to decontaminate chicken carcasses and parts (Cano et al., 2021). However, there is little information about the efficacy of common antimicrobial interventions for the control of pathogens or for shelf‐life extension of nonconventional chicken parts. Previous work from our group (Cancio et al., 2023) compared the effect of cultured dextrose fermentate, liquid buffered vinegar, and PAA on *Salmonella* reductions, background microbiota populations, and instrumental color of raw chicken livers, with PAA resulting in significantly higher reductions and minimal impact on color after sustained storage. This study compared the effectiveness of a commercial citric acid/hydrochloric acid blend (CP) (Citrilow Plus™) and sulfuric acid (SA) (Assist™) to that of PAA to reduce *Salmonella* populations and improve the shelf life of chicken hearts and livers.

## 2 MATERIALS AND METHODS

### 2.1 Inoculum preparation

Five poultry‐borne serovars of *Salmonella enterica* subsp. *enterica* (Braenderup, NVSL 96–12,528; Enteritidis, IV/NVSL 94–13,062; Hadar, JE 322 2013 MI; Heidelberg, 2247‐1; and Typhimurium, ATCC 14,028) were used in this study. A 10‐µL loopful of each strain was individually streaked onto tryptic soy agar (TSA; Remel) plates and incubated at 37°C for 24 h. A well isolated colony of each strain was picked and individually incubated in 9 mL of tryptic soy broth (TSB; Remel) for 24 h at 35°C. Cell cultures were then harvested by centrifugation at 4500 rpm for 20 min at 4°C (Frontier ^TM^ 5718R, OHAUS Corporation). The supernatant was discarded, and the cells were washed twice with 0.1% buffer peptone water (BPW; Sigma‐Aldrich) and resuspended in 10 mL of TSB. One milliliter of each strain suspension was added to 200 mL of TSB and incubated separately at 35°C for 24 h. The cell cultures were combined to make a bacterial cocktail with a final concentration of ca. 10^6^ CFU/mL, established through successive decimal dilutions in 0.1% BPW and subsequent plating onto xylose lysine deoxycholate agar (XLD; BD).

### 2.2 Sample preparation and inoculation

Chicken hearts and livers were procured from a local retail distributor in Nebraska. The products were bought frozen and kept at −20°C until further use. Samples were thawed in refrigeration at 4°C for at least 24 h. Thereafter, two 25 ± 5 g subsamples of each product type were analyzed for *Salmonella* via direct plating on XLD agar and mesophilic aerobic plate counts (APCs) using 3M APC Petri films^®^ (Neogen Corp.). Chicken heart and liver samples kept at 4°C were inoculated separately by immersion in the *Salmonella* cocktail (ca. 20°C) for 30 s, allowed to drain over a stainless‐steel grill, and air‐dried for 20 min inside a biosafety cabinet. The inoculated samples were then placed in a refrigerator at 4°C for 24 h for bacterial cold adaptation and further microbial attachment to the matrix. Prior to the application of antimicrobial treatments, two subsamples of each product type were randomly selected from each batch to determine the initial *Salmonella* counts.

### 2.3 Preparation and application of antimicrobial treatments

One‐and‐a‐half‐liter solutions of 0.05% v/v (500 ppm) PAA (Birkoside MP‐2, Birko Corp.), 2% v/v Assist (SA, Safe Foods Corp.), and 5% v/v Citrilow Plus (CP, Safe Foods Corp.) were prepared by diluting concentrated solutions in chilled (4 ± 1°C) sterile distilled water. The PAA concentration was verified using a colorimetric assay (Vacuettes kit, K‐7904C, CHEMetrics), and the pH of the other solutions was measured with a pH meter (Accumet^R^ AB150 pH/mV, Fisher Scientific). Distilled water was used as control to assess the effect of immersion and mechanical agitation at reducing bacterial populations.

Eight 25‐g samples were used per treatment (CP, PAA, SA, and water) for each of the product types (hearts and livers) and sample point combinations (0, 1, 2, and 3 storage days). The experiment was replicated three times. For each product type and treatment, two 25 ± 5 g samples of inoculated product were immersed in one of the chemical solutions or in distilled water (control) maintained at 4°C. For PAA and distilled water, the samples were immersed for 90 s, whereas for Citrilow Plus and Assist, samples were immersed for 30 s. A concentration of 0.05% (500 ppm) PAA with a contact time of 90 s is commonly used in the food industry (Cano et al., 2021). A shorter dipping time was used for Citrilow Plus and Assist to minimize their potential effect on the product quality per manufacturers’ recommendations. All treatments were applied with constant mixing at 40 rpm in a shaker incubator (SHKE6000‐7, Thermo Scientific). After samples were immersed into each treatment, excess liquid was allowed to drain for 3 min at room temperature (ca. 22°C) in a biosafety cabinet before individually vacuum packing in clear plastic bags (Multivac C200, Multivac Inc.). The samples were then stored at 4°C and subsequently used for microbial analysis as described below.

### 2.4 Microbiological analysis

Chicken livers and hearts were aseptically taken from the vacuum‐sealed bags on days 0, 1, 2, and 3 posttreatment. For each product type and treatment, two subsamples were analyzed on each day. Samples were weighed (25 ± 5 g) into a sterile stomacher bag (Whirl‐Pak^®^, Thomas Scientific LLC) and mixed with 0.1% BPW using an automatic diluter (IUL Smart Dilutor, NEU‐TEC GROUP, INC.) to attain a 1:10 ratio. Samples were then homogenized at 200 rpm for 90 s (Stomacher^®^ 400 Circulator, Seward Ltd.). The mixtures were decimally diluted, plated onto XLD agar in duplicates, and incubated at 37°C for 24 h. *Salmonella* counts were expressed as log CFU/g and the reductions calculated by the difference in average *Salmonella* counts pretreatment and at the specific sampling time points. Following the same procedure, non‐inoculated chicken hearts and livers were used in the analysis of APC by enumerating these samples on days 0, 1, 2, and 3 posttreatment using two subsamples from each product/treatment and plated onto APC Petrifilm^™^ in duplicate, followed by incubation at 35°C for 48 h. APC values were expressed as log CFU/g.

### 2.5 Color evaluation

Color analysis was conducted using the same non‐inoculated chicken heart and liver samples used for APC before plating. Objective color measurements were obtained using a portable colorimeter (Minolta CR‐300 Chroma Meter with DP‐301 Data Processor) with an 8‐mm aperture, 0° viewing angle, a D65 light source, and Hunter Lab color space. The instrument was calibrated with a standard white Minolta plate inserted in a vacuum seal bag like that used for the chicken hearts and livers to counteract the color and light reflectance properties of the packaging used (Petracci & Fletchert, 2002). All measurements were taken on three distinct surface locations of the vacuum‐sealed chicken hearts and livers that were free from noticeable defects (e.g., bruises) and the results were averaged. Tissue color measurements were taken on days 0, 1, 2, and 3 following sample treatment and values were expressed as L (lightness), a (redness), and b (yellowness).

### 2.6 Statistical analyses

Three independent replications were conducted for each product, treatment, and time combination using different batches of product and freshly prepared bacterial cocktails and antimicrobial treatments on separate days. Data (*Salmonella* reductions, APC reductions, L, a, and b) for chicken hearts and livers were analyzed separately using a two‐by‐four factorial two‐way analysis of variance. Treatment and time were analyzed as independent variables with replications as block. When there was significant difference (*p *< 0.05), Tukey–Kramer's post hoc test was applied to separate the means. All statistical analyses were conducted using SAS Version 9.4 (SAS Institute).

## 3 RESULTS AND DISCUSSION

Mean APC values for chicken hearts and livers across product batches were 3.25 ± 0.13 and 3.27 ± 0.12 log CFU/g, respectively. Background *Salmonella* was not detected in any of the three replicates at a 10‐CFU/g detection limit. For artificially inoculated samples, the average initial *Salmonella* counts were 4.75 ± 0.10 and 4.69 ± 0.10 log CFU/g for chicken hearts and livers, respectively. The average pH values of the 2% v/v Assist (SA; sulfuric acid) and the 5% v/v Citrilow Plus (CP; citric acid/hydrochloric acid blend) solutions were 0.93 and 0.66, respectively.

### 3.1 *Salmonella* reductions on chicken hearts

Compared to the water control, SA was the only treatment that significantly (*p* = 0.0133) reduced *Salmonella* populations on artificially inoculated chicken hearts immediately after application (Table 1). However, on days 1 and 2, PAA was the only treatment that showed significantly greater reductions compared to the control (*p* = 0.0013 and *p* = 0.0140, respectively). On day 3, all the treatment effects were minimal. There was no significant interaction between storage time and the antimicrobial (*p *= 0.4540). Overall, PAA showed the greatest numerical reductions of *Salmonella* populations on chicken hearts, followed by SA and CP. Although these reductions were significantly different from the control, there were no significant differences between SA and CP, suggesting that both chemicals were at least as effective as PAA. PAA was expected to show the greatest reductions given that it has already been proven effective at reducing *Salmonella* in various other raw poultry products (Bauermeister et al., 2008; Cano et al., 2021; Cano et al., 2022), including chicken livers (Cancio et al., 2023). Our results are similar to those of Moore et al. (2017), who evaluated different antimicrobials and observed the highest reductions in *Salmonella* with a 10‐s dip of 0.10% PAA solution. They reported 0.9 and 1.4 CFU/g log reductions on days 0 and 1, respectively. King et al. (2005) reported similar results with PAA (0.10%, 15 s, 45 or 55°C) used in spray post‐chill applications on beef carcasses. The authors, however, did not report significant reductions with PAA at lower concentrations (0.02% or 0.06%). This could have resulted from the high temperatures of PAA solutions used (43 ± 5°C) as compared to the chilled (4°C) solutions used in our study. Our results, on the other hand, are lower than those of Nagel et al. (2013), who reported 2.0–2.1 *Salmonella* log reductions with a 20‐s dip of 0.04% and 0.10% PAA solution (4 ± 2°C) in broiler carcasses.

**TABLE 1:** *Salmonella* reductions on chicken hearts treated with various antimicrobials and stored for up to 3 days at 4°C.

Storage time (Days)	*Salmonella* reductions (mean log CFU/g ± SE) *n* = 6			
	Water	PAA	SA	CP
0	0.95 ± 0.03^b,x^	1.34 ± 0.04^a,b,x^	1.48 ± 0.10^a,x^	1.07 ± 0.19^a,b,x^
1	0.94 ± 0.15^b,x^	1.61 ± 0.13^a,x^	1.29 ± 0.09^a,b,x^	1.27 ± 0.19^a,b,x^
2	0.96 ± 0.08^b,x^	1.49 ± 0.10^a,x^	1.35 ± 0.09^a,b,x^	1.05 ± 0.11^a,b,x^
3	0.98 ± 0.07^a,x^	1.33 ± 0.25^a,x^	1.40 ± 0.04^a,x^	1.32 ± 0.12^a,x^

*Note*: Within a row, values with a common letter superscript (abc) are not significantly different at a 5% probability level. Within a column, values with the same letter superscript (xyz) are not significantly different at a 5% probability level.

Abbreviations: CP, citric/hydrochloric acid blend (5.0%, Citrilow Plus); PAA, peroxyacetic acid (0.05% or 500 ppm); SA, sulfuric acid (2.0%, Assist); SE, standard error.

After PAA, SA was the second most effective treatment in decontaminating chicken hearts. This commercial antimicrobial is a mixture of sulfuric acid (37%–43%) and water (47%–60%), with a low concentration of sodium sulfate (0%–7%). Overall, SA resulted in 1.29–1.48 log reductions across the 3 days. PAA had numerically higher but statistically similar log reductions (1.33–1.61 CFU/g) compared to SA for all the 3 days. Scott et al. (2015) reported log reductions of 0.8–0.9 and 1.1–1.2 log CFU/mL with contact times of 10 and 20 s, respectively, when using an SA and sodium sulfate blend (SSS) at pH 1.1 on inoculated raw chicken wings. Other researchers who immersed beef cheek meat in SSS at 1.8 pH for 1–5 min reported *Salmonella* reductions ranging from 1.0 to 1.5 log CFU/cm^2^ (Schmidt et al., 2014). Geornaras et al. (2012) also conducted a study on beef trimmings using SSS (pH 1.2) with a 30‐s contact time and found reductions ranging from 0.5 to 0.7 log CFU/cm^2^. Overall, in this study, higher reductions were seen, indicating that SA at approximately pH 1 applied for 30 s may be an effective antimicrobial against *Salmonella* on chicken hearts. The effective activity of SA lies in its strong oxidative and corrosive nature, which instantly kills microorganisms even with a short exposure time (Wang et al., 2018). Low pH negatively impacts cellular structure and function increasing permeability of the cell membrane, which results in acidification of the cell contents (Lund et al., 2020; Scott et al., 2015; Tan et al., 2014).

Treatment with a proprietary blend of citric and hydrochloric acid (CP) yielded reductions ranging from 1.0 to 1.32 log CFU/g across the 3 days; though, these were not statistically different from the control or the other chemical treatments. Tan et al. (2014) reported widely varying reductions (2.92–6.52 log CFU/g) in *Salmonella* counts when using hydrochloric acid (HCl) at pH ranges of 1.2–3.8. Nevertheless, they also observed that citric acid alone (pH 2.9) inhibited *Salmonella* more effectively than HCl on chicken meat surfaces. The higher reductions in *Salmonella* populations were attributed to their dissociation constants, represented by the pKa value (citric acid 3.14), which exceed that of HCl (pKa 7.0) (Birk et al., 2010) implying a greater proportion of undissociated acid in the organic acid solutions, thereby enhancing their bactericidal activity (Narendranath et al., 2001). The undissociated acid forms easily diffuse across the cell membrane leading to the accumulation of toxic components in the form of weak acid anions in the cytoplasm of the cells (Ricke, 2003; Salmond et al., 1984). Using a commercial mixture of citric and hydrochloric acid (Chicxide, Birko Corp.), Laury et al. (2009) observed *Salmonella* reductions on broiler carcass surfaces of 1.3 log CFU/mL of rinsate with spray applications and 2.3 log CFU/mL of rinsate after immersion with 20‐s contact time in both treatments and concluded that the treatment was effective at reducing *Salmonella*.

### 3.2 *Salmonella* reductions on chicken livers

There was no significant interaction between the storage time and antimicrobial treatment (*p *= 0.5576) for chicken livers. However, in this case, the only significant difference in treatments was seen on day 2 between PAA and the water control (*p *= 0.0408), as shown in Table 2. Immediately after treatment, none of the interventions showed *Salmonella* reductions significantly greater than the control (PAA: *p *= 0.6238; CP: *p = *0.5989; SA: *p = *0.6731), making immersion in water equally effective as the chemicals to decontaminate *Salmonella*. Overall, PAA had the highest numerical reductions, followed by CP and SA. However, after 3 days of storage, only PAA and SA yielded consistent *Salmonella* reductions of at least 1 log CFU/g; hence, these two interventions may be more suitable for use in chicken livers destined for human consumption.

**TABLE 2:** *Salmonella* reductions on chicken livers treated with various antimicrobials and stored for up to 3 days at 4°C.

Storage time (Days)	*Salmonella* reductions (mean log CFU/g ± SE) *n* = 6			
	Water	PAA	SA	CP
0	0.83 ± 0.15^a,x^	1.03 ± 0.12^a,x^	1.02 ± 0.15^a,x^	1.04 ± 0.15^a,x^
1	0.87 ± 0.16^a,x^	1.08 ± 0.11^a,x^	0.92 ± 0.18^a,x^	0.94 ± 0.07^a,x^
2	0.82 ± 0.08^b,x^	1.28 ± 0.15^a,x^	0.92 ± 0.10^a,b,x^	1.17 ± 0.11^a,b,x^
3	1.10 ± 0.13^a,x^	1.10 ± 0.12^a,x^	1.09 ± 0.19^a,x^	0.96 ± 0.27^a,x^

*Note*: Within a row, values with a common letter superscript (abc) are not significantly different at a 5% probability level. Within a column, values with a common letter superscript (xyz) are not significantly different at a 5% probability level.

Abbreviations: CP, citric/hydrochloric acid blend (5.0%, Citrilow Plus); PAA, peroxyacetic acid (0.05% or 500 ppm); SA, sulfuric acid (2.0%, Assist); SE, standard error.

In the meat and poultry industry, an intervention is often considered moderately practical if its application results in at least a 1‐log reduction of the initial microbial population (Brashears & Chaves, 2017). The average *Salmonella* reductions in chicken hearts after 3 days of storage were 1.33 ± 0.25, 1.40 ± 0.04, and 1.32 ± 0.12 log CFU/g for PAA, SA, and CP, respectively (Table 1). For chicken livers the values were 1.10 ± 0.12, 1.09 ± 0.19, and 0.96 ± 0.27 for PAA, SA, and CP, respectively (Table 2). Therefore, most treatments consistently achieved the 1‐log criterion. Furthermore, considering the recommended consumer storage time for chicken giblets for human consumption is 1–2 days at 4°C (U.S. Food & Drug Administration, 2018), all these interventions could serve as effective antimicrobial measures to reduce the potential risk of *Salmonella* in nonconventional chicken parts.

### 3.3 Effect on background microbiota

There was no statistically significant interaction between storage time and the antimicrobial treatment (*p *= 0.7777) for APC values. Data for chicken hearts are presented in Table 3. On day 0, no significant differences were observed among the treatments. However, on day 2, the APC values of chicken hearts treated with PAA (*p *= 0.0056) and SA (*p *= 0.0123) were significantly lower than the control. After day 3, only the APC values of chicken hearts treated with SA were significantly lower than the control (*p *= 0.0136). Therefore, all the treatments, including the water control, were able to maintain the APC values below the lower microbiological limit for quality fresh poultry during refrigerated storage (4°C) for up to 3 days. A value of 7 log CFU/g and above would typically indicate product spoilage (Rouger et al., 2017). Normal shelf life for chicken hearts and livers in the market is 3–5 days under refrigerated storage (U.S. Food & Drug Administration, 2018). Other researchers have also studied the effect of various antimicrobials on APC and found that they are relatively effective. Mohan and Pohlman (2016) observed APC reductions in beef trimmings when using PAA and various organic acids. Mani‐López et al. (2012) reported significant reductions in APC on pork cheek meat with 1% solutions of acetic and lactic acids.

**TABLE 3:** Aerobic plate counts of chicken hearts treated with various antimicrobials and stored for up to 3 days at 4°C.

Storage time (Days)	Aerobic plate counts (mean log CFU/g ± SE) *n* = 6			
	Water	PAA	SA	CP
0	4.05 ± 0.17^a,x^	3.51 ± 0.04^a,x^	3.46 ± 0.14^a,x^	3.78 ± 0.13^a,x^
1	3.81 ± 0.22^a,x^	3.33 ± 0.16^a,x^	3.61 ± 0.04^a,x^	3.57 ± 0.15^a,x^
2	4.33 ± 0.34^a,x^	3.44 ± 0.11^b,x^	3.52 ± 0.12^b,x^	3.82 ± 0.09^a,b,x^
3	4.28 ± 0.26^a,x^	3.64 ± 0.36^a,b,x^	3.48 ± 0.11^b,x^	3.72 ± 0.10^a,b,x^

*Note*: Within a row, values with a common letter superscript (abc) are not significantly different at a 5% probability level. Within a column, values with a common letter superscript (xyz) are not significantly different at a 5% probability level.

Abbreviations: CP, citric/hydrochloric acid blend (5.0%, Citrilow Plus); PAA, peroxyacetic acid (0.05% or 500 ppm); SA, sulfuric acid (2.0%, Assist); SE, standard error.

For chicken livers (Table 4), there was no interaction between storage time and treatment (*p *= 0.2588). Immediately after treatment (day 0), no significant differences were observed among the treatments. However, on day 2, APC in chicken livers treated with PAA were significantly lower than those of both the SA (*p *= 0.0265) and the control (*p *= 0.0074). Ultimately, all treatments kept APC values below the spoilage levels during refrigerated storage for up to 3 days.

**TABLE 4:** Aerobic plate counts of chicken livers treated with various antimicrobials and stored for up to 3 days at 4°C.

Storage time (Days)	Aerobic plate counts (mean log CFU/g ± SE) *n* = 6			
	Water	PAA	SA	CP
0	3.83 ± 0.08^a,x^	3.75 ± 0.09^a,x^	3.78 ± 0.26^a,x^	3.77 ± 0.09^a,x^
1	3.86 ± 0.13^a,x^	3.68 ± 0.12^a,x^	3.86 ± 0.14^a,x^	3.73 ± 0.08^a,x^
2	3.88 ± 0.06^a,x^	3.36 ± 0.04^b,x^	3.81 ± 0.03^a,x^	3.63 ± 0.05^a,b,x^
3	3.76 ± 0.03^a,x^	3.72 ± 0.06^a,x^	3.67 ± 0.13^a,x^	3.92 ± 0.11^a,x^

*Note*: Within a row, values with a common letter superscript (abc) are not significantly different at a 5% probability level. Within a column, values with a common letter superscript (xyz) are not significantly different at a 5% probability level.

Abbreviations: CP, citric/hydrochloric acid blend (5.0%, Citrilow Plus); PAA, peroxyacetic acid (0.05% or 500 ppm); SA, sulfuric acid (2.0%, Assist); SE, standard error.

### 3.4 Objective color

Tables 5, 6, 7 show the effect of the antimicrobials on the chicken hearts’ redness (a), yellowness (b), and lightness (L). There was a treatment‐by‐storage‐day interaction for b (*p *= 0.0377) but not for a (*p *= 0.4922) or L (*p *= 0.2090). For a, significant differences were observed immediately after treatment for SA and CP and were lower throughout storage. Chicken hearts treated with PAA were less red compared to the control only on day 1. Values for b indicate yellowness; greater values are representative of discoloration. Control and PAA treated hearts had a similar or decreased b value over time and were always less than SA or CP treatments (*p* < 0.05). Regarding the L values, SA (0 h: *p *= 0.0001) and CP (0 h: *p *= 0.0004) but not PAA (0 h: *p *= 0.7082) showed significant differences from the control immediately after treatment (day 0); however, differences became insignificant by the third day of refrigerated storage (PAA: *p *= 0.9982; SA: *p *= 0.1792; CP: *p *= 0.076), which showed that the initial treatments effects on the product lightness were only temporary. In addition, the differences in chicken hearts treated with various antimicrobials were noticeable immediately after treatment, but not on day 3.

**TABLE 5:** Redness values (a) of chicken hearts treated with various antimicrobials and stored for up to 3 days at 4°C.

Storage time (Days)	Redness, a (mean ± SE) *n* = 6			
	Water	PAA	SA	CP
0	11.42 ± 1.03^a^	11.20 ± 1.01^a^	8.55 ± 0.43^b^	8.80 ± 0.88^b^
1	14.49 ± 0.36^a^	12.33 ± 0.74^b^	11.32 ± 0.48^b,c^	10.18 ± 0.75^c^
2	14.49 ± 0.14^a^	13.55 ± 0.63^a,b^	12.04 ± 0.29^b^	12.07 ± 0.33^b^
3	14.60 ± 0.24^a^	13.76 ± 0.51^a,b^	12.23 ± 0.31^b,c^	11.93 ± 0.16^c^

*Note*: Within a row, values with a common letter superscript (abc) are not significantly different at a 5% probability level.

Abbreviations: CP, citric/hydrochloric acid blend (5.0%, Citrilow Plus); PAA, peroxyacetic acid (0.05% or 500 ppm); SA, sulfuric acid (2.0%, Assist); SE, standard error.

**TABLE 6:** Yellowness values (b) of chicken hearts treated with various antimicrobials and stored for up to 3 days at 4°C.

Storage time (Days)	Yellowness, b (mean ± SE) *n* = 6			
	Water	PAA	SA	CP
0	3.37 ± 1.21^b,x^	3.22 ± 0.71^b,x^	5.49 ± 0.44^a,x^	6.27 ± 0.25^a,x^
1	1.72 ± 0.13^c,x,y^	3.13 ± 0.79^c,x^	5.02 ± 0.05^b,x^	7.12 ± 0.68^a,x^
2	1.28 ± 0.44^c,y^	2.65 ± 0.21^c,x^	6.29 ± 0.57^b,x^	6.50 ± 0.02^a,x^
3	1.65 ± 0.18^b,x,y^	1.86 ± 0.13^b,x^	6.10 ± 0.27^a,x^	6.61 ± 0.11^a,x^

*Note*: Within a row, values with a common letter superscript (abc) are not significantly different at a 5% probability level. Within a column, values with a common letter superscript (xyz) are not significantly different at a 5% probability level.

Abbreviations: CP, citric/hydrochloric acid blend (5.0%, Citrilow Plus); PAA, peroxyacetic acid (0.05% or 500 ppm); SA, sulfuric acid (2.0%, Assist); SE, standard error.

**TABLE 7:** Lightness values (L) of chicken hearts treated with various antimicrobials and stored for up to 3 days at 4°C.

Storage time (Days)	Lightness, L (mean ± SE) *n* = 6			
	Water	PAA	SA	CP
0	42.03 ± 0.60^b^	40.80 ± 0.91^b^	47.79 ± 0.93^a^	47.30 ± 1.34^a^
1	41.63 ± 2.13^b^	42.87 ± 0.45^b^	46.57 ± 1.83^a^	47.89 ± 1.37^a^
2	41.28 ± 0.81^b^	41.67 ± 1.19^b^	45.97 ± 1.06^a^	45.93 ± 0.58^a^
3	42.72 ± 0.65^a^	42.53 ± 1.80^a^	45.11 ± 1.53^a^	45.60 ± 1.34^a^

*Note*: Within a row, values with a common letter superscript (abc) are not significantly different at a 5% probability level.

Abbreviations: CP, citric/hydrochloric acid blend (5.0%, Citrilow Plus); PAA, peroxyacetic acid (0.05% or 500 ppm); SA, sulfuric acid (2.0%, Assist); SE, standard error.

Tables 8, 9, 10 present color changes in chicken livers during 3 days of storage. There was an interaction between the antimicrobial and day of storage for a (*p *= 0.0215) and L (*p *= 0.0460), but not b (*p *= 0.1300). Just like in chicken hearts, for a, significant differences were observed immediately after treatment (day 0) for SA (*p *< 0.0001) and CP (*p *< 0.0001) but not PAA (*p *= 0.6173), as compared to the control, where the SA and CP‐treated chicken livers had lower a values indicating that the livers were less red. However, all treatments were similar (*p* > 0.05) by the third day of refrigerated storage. Likewise, b values, which increased for SA‐ and CP‐ treated livers immediately after treatment, were no longer greater than the control on any other day of storage, whereas the PAA‐treated livers were similar to the control at all the time points. Concerning L values, the only significant differences observed were on day 0 posttreatment, where SA values differed significantly from PAA (*p* = 0.0130) and the control (*p* = 0.0010). However, all these differences dissipated for day one measurements. This implied that the color changes in product L due to SA application were only temporary. Just like with chicken hearts, differences in the L of meat samples were only evident immediately after treatment but not by the second and third days of refrigerated storage.

**TABLE 8:** Redness values (a) of chicken livers treated with various antimicrobials and stored for up to 3 days at 4°C.

Storage time (Days)	Redness, a (mean ± SE) *n* = 6			
	Water	PAA	SA	CP
0	15.97 ± 0.72^a,x^	14.49 ± 1.09^a,x^	8.32 ± 2.62^b,y^	9.24 ± 1.10^b,y^
1	15.81 ± 0.89^a,x^	16.53 ± 0.88^a,x^	14.58 ± 0.52^a,x^	13.95 ± 1.06^a,x^
2	17.96 ± 0.80^a,x^	15.71 ± 0.12^a,b,x^	14.17 ± 0.58^b,x^	15.15 ± 0.29^a,b,x^
3	17.25 ± 0.90^a,x^	16.63 ± 0.32^a,x^	14.52 ± 0.64^a,x^	14.82 ± 0.59^a,x^

*Note*: Within a row, values with a common letter superscript (abc) are not significantly different at a 5% probability level. Within a column, values with a common letter superscript (xyz) are not significantly different at a 5% probability level.

Abbreviations: CP, citric/hydrochloric acid blend (5.0%, Citrilow Plus); PAA, peroxyacetic acid (0.05% or 500 ppm); SA, sulfuric acid (2.0%, Assist); SE, standard error.

**TABLE 9:** Yellowness values (b) of chicken livers treated with various antimicrobials and stored for up to 3 days at 4°C.

Storage time (Days)	Yellowness, b (mean ± SE) *n* = 6			
	Water	PAA	SA	CP
0	6.39 ± 1.24^c^	7.70 ± 0.56^b,c^	10.87 ± 1.16^a^	9.90 ± 1.26^a,b^
1	6.35 ± 0.75^a^	6.45 ± 0.78^a^	7.14 ± 0.29^a^	6.79 ± 0.49^a^
2	6.48 ± 0.43^a,b^	5.04 ± 0.53^b^	8.12 ± 0.76^a^	6.67 ± 0.19^a,b^
3	5.49 ± 0.54^a^	6.90 ± 0.33^a^	7.33 ± 1.32^a^	6.85 ± 0.19^a^

*Note*: Within a row, values with a common letter superscript (abc) are not significantly different at a 5% probability level.

Abbreviations: CP, citric/hydrochloric acid blend (5.0%, Citrilow Plus); PAA, peroxyacetic acid (0.05% or 500 ppm); SA, sulfuric acid (2.0%, Assist); SE, standard error.

**TABLE 10:** Lightness values (L) of chicken livers treated with various antimicrobials and stored for up to 3 days at 4°C.

Storage time (Days)	Lightness, L (mean ± SE) *n* = 6			
	Water	PAA	SA	CP
0	43.65 ± 2.92^b,x^	45.51 ± 0.87^b,x^	51.72 ± 1.81^a,x^	46.90 ± 2.16^a,b,x^
1	41.95 ± 1.87^a,x^	42.60 ± 1.13^a,x^	43.39 ± 1.06^a,y^	38.37 ± 1.07^a,y^
2	43.53 ± 0.61^a,x^	40.43 ± 2.07^a,x^	43.67 ± 1.47^a,y^	40.09 ± 0.76^a,y^
3	41.86 ± 1.30^a,x^	43.91 ± 0.56^a,x^	42.72 ± 1.08^a,y^	40.48 ± 0.44^a,y^

*Note*: Within a row, values with a common letter superscript (abc) are not significantly different at a 5% probability level. Within a column, values with a common letter superscript (xyz) are not significantly different at a 5% probability level.

Abbreviations: CP, citric/hydrochloric acid blend (5.0%, Citrilow Plus); PAA, peroxyacetic acid (0.05% or 500 ppm); SA, sulfuric acid (2.0%, Assist); SE, standard error.

Other researchers have observed similar temporary color effects in chicken parts when using antimicrobials containing PAA or SA. Scott et al. (2015) also reported greater b values immediately after treatment when treating chicken wings with an antimicrobial blend containing SA and sodium sulfate. The color differences were no longer significant after 24 h of storage at 4°C. Similar color changes were reported by Bauermeister et al. (2008) when they evaluated the effect of various levels (0.01% and 0.02%) of PAA on chicken carcasses. The researchers detected small differences in a and b values after 24 h of storage; however, no differences in L values were reported at that same time point.

### 3.5 CONCLUSIONS

The differences in *Salmonella* reductions due to immersion of chicken livers in PAA, SA, or CP were not significant as compared to immersion in distilled water immediately after treatment. PAA reductions became significant on the second day of treatment, but these reductions were not different from those of SA or CP, indicating that these three antimicrobials have relatively similar effectiveness against *Salmonella*. Contrary to chicken livers, *Salmonella* reductions observed when chicken hearts were immersed in SA showed significant differences immediately after treatment as compared to immersion in distilled water. Overall, all antimicrobial agents resulted in consistent reductions greater than one log in *Salmonella* counts on both chicken hearts and livers, a result that differed when distilled water was used. Furthermore, all interventions proved efficacious in minimizing the proliferation of mesophilic aerobic microflora throughout a refrigerated storage period of up to 3 days. Lastly, no significant differences color differences were detected on the third day of storage in either of the chicken meat products. The results of this study indicated that SA and CP as immersion treatments at approximately pH 1 may be effective antimicrobial interventions in chicken hearts and livers. When SA (2% v/v) and CP (5% v/v) were evaluated against PAA (0.05% or 500 ppm), they demonstrated at least equivalent efficacy. Hence, these treatments may be employed in the poultry industry as integral components of a multifaceted approach to mitigate *Salmonella* in nontraditional chicken parts.

## AUTHOR CONTRIBUTIONS

**Emma Nakimera**: Writing—original draft; methodology; writing—review and editing; formal analysis; investigation; visualization; validation. **Leslie Pearl M. Cancio**: Writing—review and editing; methodology; formal analysis. **Gary A. Sullivan**: Writing—review and editing; conceptualization. **Raziya Sadat**: Writing—review and editing; investigation. **Byron D. Chaves**: Conceptualization; funding acquisition; writing—review and editing; project administration; formal analysis; supervision; validation; methodology.

## CONFLICT OF INTEREST STATEMENT

The authors declare no known conflicts of interest. There are no known affiliations or financial interests that could compromise the study's objectivity.

## Data Availability

The data that support the findings of this study are available from the corresponding author upon reasonable request.
